# Multidrug resistance in pathogens of community-acquired urinary tract infections in Turkey: a multicentre prospective observational study

**DOI:** 10.55730/1300-0144.5641

**Published:** 2023-02-09

**Authors:** İrfan ŞENCAN, Oğuz KARABAY, Fatma Aybala ALTAY, Serap SÜZÜK YILDIZ, Hüsniye ŞİMŞEK, Melih Gaffar GÖZÜKARA, Semanur KUZİ, Gülden ESER KARLIDAĞ, Şafak KAYA, Gülnur KUL, Emine TÜRKOĞLU, Büşra ERGÜT SEZER, Nesibe KORKMAZ, Sibel YILDIZ KAYA, Merve Sefa SAYAR, Dilek BULUT, Fethiye AKGÜL, Yasemin ÇAĞ, Canan AĞALAR, Zehra BEŞTEPE DURSUN, Meltem TAŞBAKAN, Sabire Şöhret AYDEMİR, Derya SEYMAN, Mustafa YILDIRIM, Zafer HABİP, Nilgün ALTIN, Hanife UZAR, Begüm BEKTAŞ, Derya ÖZTÜRK ENGİN, Hüseyin Aytaç ERDEM, Serkan SÜRME

**Affiliations:** 1 Department of Infectious Diseases and Clinical Microbiology, Dışkapı Yıldırım Beyazıt Training and Research Hospital, University of Health Sciences, Ankara, Turkey; 2 Department of Infectious Diseases and Clinical Microbiology, Faculty of Medicine, Sakarya University, Sakarya, Turkey; 3 Department of Infectious Diseases and Clinical Microbiology Faculty of Medicine, Lokman Hekim University, Ankara, Turkey; 4 Department of Microbiology Reference Laboratory and Biological Products, General Directorate of Public Health, Republic of Turkey Ministry of Health, Ankara, Turkey; 5 Department of Communicable Diseases, Ankara Sincan Provincial Health Directorate, Republic of Turkey Ministry of Health, Ankara, Turkey; 6 Department of Infectious Diseases and Clinical Microbiology, Ünye State Hospital, Republic of Turkey Ministry of Health, Ordu, Turkey; 7 Department of Infectious Diseases and Clinical Microbiology, Fethi Sekin City Hospital, University of Health Sciences, Elazığ, Turkey; 8 Department of Infectious Diseases and Clinical Microbiology, Gazi Yaşargil Research and Training Hospital, University of Health Sciences, Diyarbakır, Turkey; 9 Department of Infectious Diseases and Clinical Microbiology, Kırıkhan State Hospital, Republic of Turkey Ministry of Health, Hatay, Turkey; 10 Department of Infectious Diseases and Clinical Microbiology, Faculty of Medicine, Gaziosmanpaşa University, Tokat, Turkey; 11 Department of Infectious Diseases and Clinical Microbiology, Çorlu State Hospital, Republic of Turkey Ministry of Health, Tekirdağ, Turkey; 12 Department of Infectious Diseases and Clinical Microbiology, Kahramankazan State Hospital, Republic of Turkey Ministry of Health, Ankara, Turkey; 13 Department of Infectious Diseases and Clinical Microbiology, Sungurlu State Hospital, Republic of Turkey Ministry of Health, Çorum, Turkey; 14 Department of Infectious Diseases and Clinical Microbiology, Van Research and Training Hospital, Republic of Turkey Ministry of Health, Van, Turkey; 15 Department of Infectious Diseases and Clinical Microbiology, Batman Research and Training Hospital, Republic of Turkey Ministry of Health, Batman, Turkey; 16 Department of Infectious Diseases and Clinical Microbiology, Faculty of Medicine, İstanbul Medeniyet University, İstanbul, Turkey, Fenerbahçe University, İstanbul, Turkey; 17 Department of Infectious Diseases, Fenerbahçe University, İstanbul, Turkey; 18 Department of Infectious Diseases and Clinical Microbiology, Kayseri Medicine Faculty, University of Health Sciences, Kayseri, Turkey; 19 Department of Infectious Diseases and Clinical Microbiology, Faculty of Medicine, Ege University, İzmir, Turkey; 20 Department of Microbiology and Clinical Microbiology, Faculty of Medicine, Ege University, İzmir, Turkey; 21 Department of Infectious Diseases and Clinical Microbiology, Antalya Education and Research Hospital, University of Health Sciences, Antalya, Turkey; 22 Department of Infectious Diseases and Microbiology, İstanbul Training and Research Hospital, University of Health Sciences, İstanbul, Turkey; 23 Department of Medical Microbiology, Faculty of Medicine, İstanbul Medeniyet University, İstanbul, Turkey; 24 Department of Infectious Diseases and Clinical Microbiology, Viranşehir State Hospital, Republic of Turkey Ministry of Health, Şanlıurfa, Turkey; 25 Department of Infectious Diseases and Clinical Microbiology, Sancaktepe İlhan Varank Training and Research Hospital, Republic of Turkey Ministry of Health, İstanbul, Turkey; 26 Department of Infectious Diseases and Clinical Microbiology, Haseki Training and Research Hospital, Republic of Turkey Ministry of Health, İstanbul, Turkey; 27 Department of Medical Microbiology, Institute of Graduate Studies, İstanbul University-Cerrahpasa, İstanbul, Turkey

**Keywords:** Community-acquired infection, urinary tract infections, multidrug resistance, quinolone resistance

## Abstract

**Background/aim:**

To have country-wide information about multidrug resistance (MDR) in isolates from community-acquired urinary tract infections (CAUTI) of Turkey, in terms of resistance rates and useful options.

**Materials and methods:**

We used a geocode standard, nomenclature of territorial units for statistics (NUTS), and a total of 1588 community-acquired isolates of 20 centres from 12 different NUTS regions between March 2019 and March 2020 were analysed.

**Results:**

Of the 1588 culture growths, 1269 (79. 9%) were *Escherichia coli* and 152 (9.6%) were *Klebsiella* spp. Male sex, advanced age, and having two or more risk factors showed a statistically significant relation with MDR existence (p < 0.001, p: 0.014, p < 0.001, respectively) that increasing number of risk factors or degree of advancing in age directly affects the number of antibiotic groups detected to have resistance by pathogens. In total, MDR isolates corresponded to 36.1% of our CAUTI samples; MDR existence was 35.7% in *E. coli* isolates and 57.2% in *Klebsiella* spp. isolates. Our results did not show an association between resistance or MDR occurrence rates and NUTS regions.

**Conclusion:**

The necessity of urine culture in outpatient clinics should be taken into consideration, at least after evaluating risk factors for antibacterial resistance individually. Community-acquired UTIs should be followed up time- and region-dependently. Antibiotic stewardship programmes should be more widely and effectively administrated.

## 1. Introduction

Urinary tract infections are one of the most commonly seen infections in clinical practice. They are not only seen in hospitalised patients, but among “out-of-hospital” population as well [1]. Depending on the current changes at susceptibility patterns of the pathogens, guidelines are being updated regularly.

Infections due to resistant microorganisms are seen even in patients who have no connection to healthcare [2,3].

Making the right choices for empirical treatment of these infections in outpatient clinics is an important subject and it requires available information about the categorization of the infection, common pathogens, and antibiotic susceptibility profiles of these microorganisms. A successful revelation about current distribution of pathogen microorganisms and their susceptibility patterns in community-originated, not-healthcare-associated urinary tract infections would directly lead to optimizing empirical treatment schedules of these infections in every line of healthcare.

Such information would also make an important contribution to antibiotic stewardship not only by avoiding ineffective treatments and their side effects but by helping to struggle with antibiotic resistance and higher treatment costs as well.

This study was planned as a multicentre prospective observational study to have a country-wide information about MDR resistance in community-acquired urinary tract infections (CAUTI).

According to our literature survey, our study is the first wide distributed and NUTS-regions-oriented search about MDR pathogens in CAUTI isolates of Turkey.

## 2. Materials and methods

### 2.1. Study design and sample collection

Patients were recruited from infectious diseases, emergency, urology, internal medicine, family medicine, pregnancy follow-up policlinics and clinics and general district policlinics of hospitals as well from all over Turkey. Nomenclature of territorial units for statistics (NUTS) is a geocode standard for referencing the subdivisions of countries for statistical purposes^1^. It is used by European Union and candidate countries and was defined for Turkey in 2002 in agreement between and the Turkish authorities^2^. Population size, geographical features, regional development plans, essential statistical indicators and socioeconomic development sorting are used to define these units^3^.

To obtain an even distribution of pathogens and susceptibility profiles, 21 centres from 12 different NUTS regions were chosen. Infectious disease specialists working in secondary or tertiary care facilities from these provinces had been got in touch and those who accepted to join our study fulfilled the formal requirements of ethics committee. University of Health Sciences Dışkapı Yıldırım Beyazıt Research and Training Hospital Clinical Researches Ethics Committee had given consent to our study (Date: 04.02.2019, Number: 59/04).

All these centres had given permission to use samples, laboratory findings, and clinical features data of their patients in our study. All patients were informed and their consents were taken by the participant doctor before interviewing them and also using their test results.

To avoid the misinterpretation of the results, we firstly and carefully used the definitions those are present in textbooks, current guidelines, or international expert initiatives [4–6]. Subsequently, we chose the right questions to determine the study population, those actually having community-acquired infection (below). We set out the optimum scale of antibiotics to be investigated, both representing the groups and eligible to study in local laboratories.

We admitted the threshold of 20% to evaluate whether an agent is appropriate or not for the treatment of CAUTI, as the resistance prevalence at which an antibiotic is no longer recommended for empirical treatment of acute cystitis is declared such [4].

The specimens were obtained between 01.03.2019 and 01.03.2020. The representation of 12 NUTS regions by at least 250 samples for each region was planned to obtain 95% confidence interval. Twenty of the 21 chosen centres had sent us the samples while one could not do so. We recruited the samples of patients which had the following characteristics. These characteristics are defining a patient who has no relation with hospitals or healthcare recently, so having a “community-acquired” infection and they are set coherently with guidelines or text books [7,8].

-those over 18 years,
-those who had no current hospitalisation or nursing home residency,
-those who currently (minimum for the last 48 h) had no constant or intermittent urinary catheterisation,
-those who had no regular visits to hospital/healthcare unit (for dialysis, wound dressing etc.) in the last 30 days,
-those who had no antibiotic use for any purposes in the last 1 month period,
-those who had not been diagnosed with urinary tract infection two or more times in the past year before admission,
-and those who had given consent to participate after being informed about the study.

These criteria were checked by a standardized questionnaire for the outpatients who had been suspected as urinary tract infection and whose middle flow urine sample analysis showed at least 10 leukocytes per millilitre confirmatively.

Patients who fully met the criteria were then informed and their consents were obtained. Their systemic investigation, physical examination findings, and risk factors were noted in a standardized form. Criteria for upper urinary tract infection and lower urinary tract infection were determined according to the current literature and guidelines [4–6]. Urine cultures were performed according to national microbiology standards in all participating centres. Grown microorganisms were identified and antibiotics susceptibility tests were held by using European Committee on Antimicrobial Susceptibility Testing (EUCAST) breakpoint tables (Version 9.0, 2019) which had been valid in the time interval of the study. The results of identification and antibiograms were also recorded to the standardized form afterwards. Isolated bacteria and the records of the patients were sent to our hospital for data collection and microbiological confirmation at a reference centre (Turkish Public Health Institute). Stuart medium was used for the transport of the bacteria. All the samples and forms were given the same codes per cases and these codes were used in all procedures afterwards.

### 2.2. Processing of samples and interpretation of the results

The isolated bacteria were collected and stored at 2–6 °C for maximum 4 weeks before transporting to our hospital. We collected all the samples at −20 °C for maximum 3 months before delivering them to Turkish Public Health Institute, where microbiological confirmation would be made by MALDI-TOF (Bruker Microflex LT, Germany) and antibiotic susceptibility tests would be performed with disc diffusion test by the EUCAST standards. Figure shows all of the steps of our study as a flow chart.

Multidrug-resistant bacteria (MDR) were defined as bacteria nonsusceptible to at least one agent each in three or more antimicrobial categories. As a key point, intrinsic resistance was not addressed while assessing MDR situation of the microorganisms, just as recommended by authors [9].

### 2.3. Statistical analysis

Statistical analysis was made by using SPSS 23.0 (IBM SPSS Inc., Chicago, IL, USA) programme. Conformity of the variables to normal distribution was checked by using probability pattern histograms and graphics and performing the Kolmogorov–Smirnov test. Demographic data were presented as numbers and percentages (%), mean and standard deviation (±), median and minimum-maximum.

Pearson chi-squared test and Fisher’s exact test were used for categorical values. Variables that provided statistically significant results in chi-squared test were taken under binary logistic regression test by using “enter” method. A multicollinearity status was determined between age and UTI risk factors in tests performed while modelling, so these independent variables were tested with backward eliminating method. The results were considered statistically significant with a p value of <0.05 and by 95% confidence interval.

## 3. Results and discussion

### 3.1. Background

There are a few and mostly local studies about the resistance patterns of urinary pathogens in Turkey. Some of them are about MDR pathogens in CAUTIs, but they are restricted to one or a few geographic areas. Alternatively, there are some studies comprehending wider range of areas in Turkey, but the studied object is not MDR pathogens, generally ESBL producing ones [10–13]. Our study is the first wide distributed search about MDR pathogens in CAUTI isolates of Turkey.

When we searched the literature generally, we saw that there are many studies on CAUTI which investigate risk factors, resistance of the pathogens, useful antibiotic choices, etc. The results vary in different studies due to not only conditional and designing differences of the studies but the differences in definitions as well. We observed that it is very difficult to elect the real community-acquired UTI’s and to rule out those related to healthcare.

Moreover, different MDR definitions are suggested in different studies since multiple group resistance patterns have begun to be observed.

We are assured that our results are actually rendering CAUTIs because all the definitions and criteria are defined with regard to textbooks and guidelines on this subject strictly and consistently, as mentioned above in the materials and methods section.

### 3.2. About demographics and patient characteristics

Fifteen hundred and eighty-eight samples gathered from 1588 UTI cases and asymptomatic bacteriuria of pregnant women meeting the criteria were recruited to the study (one sample per one patient; 1304 (82.1%) were female and 284 (17.9%) were male. The median age was 48 years (18–102), while the mean was 48.69 (±20.28).

When the distribution of the patients’ symptoms and signs were examined, top three were dysuria, pollakiuria, and urgency (as shown in Table 1).

Eight hundred and seven (51.1%) of the patients were admitted with signs and symptoms just related to lower UTI, while 15 (0.9%) had those related to upper UTI and 650 (41.1%) showed those related to both of them. Besides, 102 (6.4%) patients had asymptomatic bacteriuria. The signs of sepsis (hypotension, cyanosis of fingers, and altered state of consciousness) were assessed in upper UTI signs in statistical analysis (as shown in Table 1).

### 3.3. MDR and risk factors

Of the 1588 growths, 1269 (79.9%) were *E. coli* and 152 (9.6%) were *Klebsiella* spp. Ninety-six isolates were gram-positive (6.04%) and 32 were nonfermentative gram-negative (2%) as 30 *Pseudomonas* spp. and 2 *Acinetobacter* spp. strains. Nonfermentative gram-negative bacteria were not included in MDR analysis because their numbers were small.

We investigated pregnancy, diabetes mellitus, urolithiasis, benign prostate hyperplasia, menopause, genital cancer, prostate CA, and history of urinary tract surgery as the risk factors for UTI. Five hundred and fifty (34.8%) patients did not have any of these, while 325 (20.6%) had only one risk factor, and 706 (44.7%) had two or more (as shown in Table 1).

We found that the presence of MDR is associated with sex and having two or more of the risk factors above. This association was statistically significant (p < 0.001 and p: 0.006, respectively). Use of any antibiotic for any reason in the last 90 days was also found to effect the antibiotic resistance, but the relation was not statistically significant (p: 0.062).

We classified the growings as those having MDR or those not having MDR to investigate if MDR existence is associated with any of the descriptive characteristics of the patients: male sex, advanced age, and having two or more risk factors showed a statistically significant relation with MDR existence (p < 0.001, p: 0.014, p < 0,001, respectively) (as shown in Table 2).

In our study, extension of the resistance was found to be associated with sex and associated with increasing number of the risk factors. This association was statistically significant (p < 0.001 and p: 0.006, respectively). Use of any antibiotic for any reason in the last 90 days was not found to affect the antibiotic resistance, in meaning of statistical significance (p: 0.080 for existence of MDR). Statistical analysis between subgroups showed that increasing number of risk factors or degree of advancing in age directly affects the number of antibiotic groups detected to have resistance by pathogens (as shown in Table 2).

When urinary tract infection risk factors were assessed for MDR existence by logistic regression analysis with backward elimination method, one unit increase in age led to 1.007-fold [(1.001–1.013), p < 0.01] increase in favour of MDR existence. Moreover, MDR existence had an odds ratio of 1.72 (1.27–2.34) and 1.71 (1.18–2.48) by the presence of male sex and urolithiasis, respectively (as shown in Table 3).

In total, MDR isolates corresponded to 36.1% of our CAUTI samples, MDR existence was 35.7% in *E. coli* isolates and 57.2% in *Klebsiella* spp. isolates. There is not a prior study giving countrywide results about rates of MDR *E. coli* or *Klebsiella* spp. in CAUTIs, but our results are consistent with those of Köksal et al.’s study regarding to Turkey’s data in SMART 2011–2012, consisting of 363 gram-negative growings from 6 centres and showing an ESBL-positive *E. coli* rate of 38% and and ESBL-positive *Klebsiella* spp. rate of 41.7% in CAUTIs [13]. There are also some study results from sole centres like Kurutepe et al.’s and Güçlü et al.’s studies. Kurutepe et al. found that 1203 isolates from a sole centre, which refers to TR3 NUTS region in our study, had showed a mean MDR *E. coli* rate of 24.5% in 5 years (1998–2003) [10]. Our results which had come from TR3 NUTS region showed an MDR rate of 27% in only 63 samples. Also, a recent report from Güçlü et al. showed that the MDR rate of 240 samples from one centre between January 2017 and July 2019 was 53%, and this result also points out higher MDR rates [12]. These results are consistent with those of our study and give an idea about how serious the antibiotic resistance problem is.

We have to remember that these prior studies are not standard in the meaning of compliance with CAUTI criteria; in fact, some studies had assumed outpatients directly as having community-originated infections, besides some more strictly measuring ones.

The lowest MDR rate was observed in samples from NUTS-9 region and the highest rate was in those from NUTS-2 and NUTS-6 (50.0% and 49.4%, respectively). Our results did not show an association between resistance or MDR occurrence rates and NUTS regions. The striking fact is that, except NUTS-9 region (8.4%), MDR rates in CAUTIs are all above 25% in Turkey (as shown in Table 4).

Provinces from NUTS-9 region have lower antibiotic percentages in prescriptions when compared to Turkey’s mean results [14]. The province which we had chosen to represent NUTS-9 region in our study was also declared to have the lowest antibiotic percentages in prescriptions in year 2017 in that study. These two results are supporting the role of antibiotic overuse in existence of MDR pathogens. However, it is not a valid argument when we analyse NUTS-2 region which has also low antibiotic prescription rates but higher MDR results. Intense use of antibiotics in primary care is accepted as a risk factor for resistance development in bacteria, but as seen with these results it is not sufficient alone to explain the consequence. Even though we were not able to show the association between antibiotic use in the last 90 days and MDR existence statistically, an association is seemed to occur in our study, too (Table 2).

### 3.4. Which antibiotics can be recommended in this trouble?

Cefuroxime axetil, nitrofurantoin, and gentamicin were antibiotics with the resistance rate under 20% for *E. coli* while cefuroxime axetil and gentamicin were the same for *Klebsiella* spp. Unfortunately, ciprofloxacin, TMP/SMX, fosfomycin, and cefixime had resistance rates of 20%–30% for both of them (Table 5).

Cefuroxime axetil, nitrofurantoin, and gentamicin were the options with resistance rates under 20% when the isolates were totally analysed.

The analysis which was made on only MDR *E. coli* isolates showed that 37 of 434 (8.5%) isolates were fosfomycin-resistant, while 166 of 407 (40.8%) were nitrofurantoin-resistant (as shown in Table 6). This result resembles that of Demir and Büyükgüçlü’s study, which determines high fosfomycin sensitivity (88%) in one region, 555 ESBL producing *E. coli* and *Klebsiella* strains, in a period of four years between 2008 and 2012 [15].

We also made an analysis on patients who had only lower urinary tract symptoms, to guide empirical treatment of cystitis in outpatient clinics and the results were as follows: 46 samples from 805 patients (5.7%) resulted as resistant isolates to nitrofurantoin and 151 from 528 (28.6%) were resistant to fosfomycin (Table 7). Nitrofurantoin seems useful for empirical treatment of lower UTI, while fosfomycin is not.

### 3.5. Ciprofloxacin resistance and MDR existence

Quinolone resistance in CAUTIs is a worldwide problem [16–19]. Studies of the last two decades from our country show similar results. In the multicentre study of Arslan et al., ciprofloxacin resistance was %17 in uncomplicated UTIs and 38% in complicated UTIs, in isolates of the year 2004, which were mostly *E. coli* [11]. An İstanbul study on outpatient isolates of *E. coli* showed the increase of ciprofloxacin resistance from 17% to 43% between 2014 and 2018 [20]. These studies do not have standard definitions for CAUTIs. Overall, ciprofloxacin resistance was detected as 20.4% in *E. coli* and 21.3% in *Klebsiella* spp. in our study after two decades (as shown in Table 5). The definition used is important when interpreting the resistance results, like differentiating outpatients and real community-originated patients.

Like many other countries, empirical quinolone use is common in emergency services and primary care facilities and they have been usually preferred as first choice for treatment of urinary tract infections with prescription rates up to 50%–70% in Turkey [21–24]. Not surprisingly, steadily increasing quinolone resistance have been detected in the last two decades parallel to overuse [11, 20, 21, 25].

Furthermore, quinolone resistance had been associated with multidrug resistance [11, 26, 27]. In our study, ciprofloxacin resistance was found as 48.6% in MDR (+) *E. coli* and 36.8% in MDR positive *Klebsiella* spp. and confirms this suggestion (as shown in Table 8).

Overall, ciprofloxacin resistance reached 20%, so efforts should be focused to stop quinolone use as first choice in empirical treatment of CAUTI. Especially in patients with male sex, advanced age or more than two risk factors in background, quinolone use should be thought twice before setting out the treatment. A preliminary culture could be useful.

### 3.6. Limitations of the study

We targeted to gather 250 samples per a NUTS region to obtain powerful representation of the population, but unfortunately, we were able to collect only 1588 samples, with very low amounts from NUTS-4, NUTS-7, and NUTS-TRA regions. Also NUTS-1 region, which is completely covered by İstanbul with 15 million population could not be investigated with enough sample size. Even so, this is the first study performed to see the complete picture with participation of all NUTS regions of Turkey with different demographical, industrial, and socio-economic characteristics. We hope that new and more comprehensive studies could be realized on this ground in the future.

## 4. Conclusion

In total, MDR isolates corresponded to 36.1% of the samples. MDR rates in CAUTIs are above 25% in all NUTS regions of Turkey. This means, by the most optimistic prediction, one of every three or four CAUTI patients has MDR pathogens in urine and cannot be effectively treated with routine empirical treatments. These results also suggest that the necessity of urine culture in outpatient clinics should be taken into consideration, at least after evaluating risk factors for antibacterial resistance individually.

Community-acquired UTIs are not innocuous and should be followed up-time and region-dependently.

As the last words, antibiotic stewardship programmes should be more widely and effectively administrated, primarily by means of education.

## Figures and Tables

**Figure:**
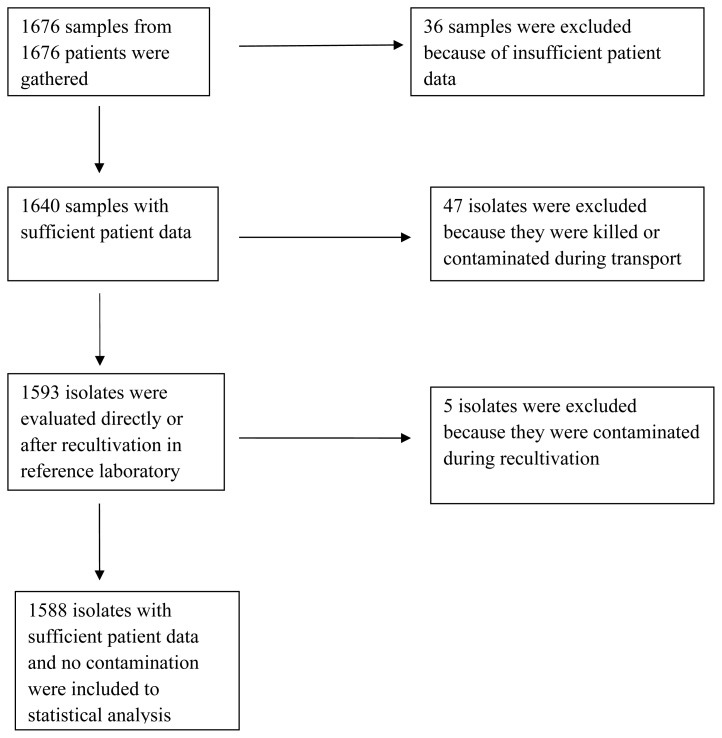
Flow chart of the study.

**Table 1:** Distribution of symptoms and signs of the patients.

	Present (n, %)	Total (n,%))
**Symptoms and signs related to UTI**		
Dysuria	1313 (83.3)	1576 (100)
Urgency	866 (54.8)	1580 (100)
Pollakiuria	1142 (72.4)	1578 (100)
Suprapubic tenderness	816 (51.7)	1577 (100)
Flank pain	428 (27.1)	1578 (100)
Chills	375 (23.8)	1578 (100)
Malaise	368 (23.4)	1588 (100)
Perineal-pelvic pain	100 (7.5)	1342 (100)
Fever (as a symptom)	289 (18.3)	1578 (100)
Fever (>38 °C by physical examination)	258 (16.3)	1580 (100)
Tachycardia	157 (10.0)	1575 (100)
Costovertebral angle tenderness	285 (18.1)	1577 (100)
Hypotension	68 (4.3)	1575 (100)
Altered state of consciousness	30 (1.9)	1575 (100)
Cyanosis of fingers	7 (0.4)	1574 (100)
Those having only lower UTI signs and symptoms	807 (51.1)	1580 (100)
Those having only upper UTI signs and symptoms	15 (0.9)	1580 (100)
Those having both lower and upper UTI signs and symptoms	650 (40.9)	1580 (100)
Those having no signs or symptoms of UTI	102 (6.4)	1580 (100)
**UTI risk factors**		
Pregnancy	223 (17.2)	1295 (100)
Diabetes mellitus	221 (14.0)	1576 (100)
Urolithiasis	160 (10.2)	1572 (100)
Benign prostate hyperplasia	145 (51.4)	282 (100)
Menopause	377 (29.0)	1292 (100)
Genital cancer	20 (1.3)	1519 (100)
Prostate cancer	17 (6)	282 (100)
Past urinary tract surgery	66 (4.2)	1568 (100)
Those having no risk factor for UTI	550 (34.8)	1581 (100)
Those having only one risk factor for UTI	325 (20.6)	1581 (100)
Those having two or more risk factors for UTI	706 (44.7)	1581 (100)

UTI: Urinary tract infection.

**Table 2:** Comparison of some demographic and risk factors related to presence of MDR.

	MDR−	MDR +	p
**Sex (n=1548)**			
Female** (n=1266)	848 (67.0)	418 (33.0)	**<0.001** *
Male (n=282)	144 (51.1)	138 (48.9)	
**Age (n=1517)**			
18–65	753 (65.8)	392 (34.2)	**0.014** **
66–79	159 (58.2)	114 (41.8)	
80–102	55 (55.6)	44 (44.4)	
**Antibiotic use in the last 90 days (n=1539)**			
None (n=1334)	865 (64.8)	469 (35.2)	0.080*
Present (n=205)	120 (58.5)	85 (41.5)	
**UTI risk factors (n=1549)**			
Less than two (n=1237)	822 (66.5)	415 (33.5)	**<0.001** *
Two or more (312)	171 (54.8)	141 (45.2)	

MDR: multidrug resistance; UTI: urinary tract infection.

* Pearson chi-squared test.

** Statistically significant difference was determined between age 18–65 and age 66–79 (**p = 0.020**) and between age 18–65 and age 80–102 (**p = 0.042**) in in-group pairwise comparisons. (The difference in numbers is due to the difference in numbers of comparable data.)

*** Because *P. aeruginosa* was not included in MDR calculation and they were all gathered from women, the number of women is 1266 in these statistics.

**Table 3:** Stepwise logistic regression of demographic and risk factors related to presence of MDR.

p-value							Odds ratio (Exp B)-CI	n=1520
Step 1	Step 2	Step 3	Step 4	Step 5	Step 6	Step 7		
								**MDR existence**
0.163	0.155	0.096	0.062	0.053	0.010	0.014	**1.007 (1.001–1.013)**	**Age**
0.119	0.119	0.088	0.003	0.001	0.001	0.001	**1.728 (1.272–2.348)**	**Sex** *
0.169	0.168	0.171	0.188	0.194	Eliminated	Eliminated	**-**	Pregnancy
0.999	Eliminated	Eliminated	Eliminated	Eliminated	Eliminated	Eliminated	**-**	Diabetes mellitus
0.006	0.006	0.006	0.006	0.007	0.004	0.004	**1.717 (1.188–2.482)**	**Urolithiasis**
0.395	0.394	0.378	Eliminated	Eliminated	Eliminated	Eliminated	**-**	BPH
0.876	0.875	Eliminated	Eliminated	Eliminated	Eliminated	Eliminated	**-**	Menopause
0.131	0.131	0.131	0.128	0.124	0.131	Eliminated	**-**	Genital cancer
0.316	0.315	0.310	0.326	Eliminated	Eliminated	Eliminated	**-**	Prostate cancer
0.056	0.056	0.057	0.062	0.044	0.041	0.064	**-**	Urinary surgery

MDR: multidrug resistance; BPH: benign prostate hyperplasia.

* Female sex is reference category; SE: standardized error; CI: confidence interval.

**Table 4:** The NUTS regions distribution of samples and MDR bacteria.

	n (%*)	MDR +, n (%**)
**NUTS-1**	91 (5.7)	39 (42.9)
**NUTS-2**	123 (7.7)	58 (50.0)
**NUTS-3**	63 (4.0)	17 (27.4)
**NUTS-4**	9 (0.6)	3 (33.3)
**NUTS-5**	103 (6.5)	35 (34.0)
**NUTS-6**	175 (11.0)	84 (49.4)
**NUTS-7**	15 (0.9)	6 (40.0)
**NUTS-8**	205 (12.9)	84 (42.0)
**NUTS-9**	251 (15.8)	21 (8.4)
**NUTS-TRA**	7 (0.4)	2 (28.6)
**NUTS-TRB**	339 (21.3)	123 (36.5)
**NUTS-TRC**	207 (13.0)	89 (44.7)

NUTS: nomenclature of territorial units for statistics MDR: multidrug resistance.

* percent for column,

** percent for line, analysis on 1566 MDR positive samples excluding *Pseudomonas* spp.

**Table 5:** Percentages and resistance rates of isolated bacteria for the routinely used antibiotics.

*Bacteria (%* ^*^ *)*	AM(n,%)	AMC (n,%)	P(n,%)	CZ(n,%)	CXA(n,%,)	CFM(n,%)	TMP-STX(n,%)	FF(n,%)	F(n,%)	GEN(n,%)	CIP(n,%)	VA(n,%)	≥2res(n,%)	MDR
***E. coli*** ***n=1269 (79.9)***	**622 (49.8)** ***n:1248***	**285 (24.3) ** ***n:1175***	**N/A**	**635 (91.5)** ***n:694***	**159 (13.6) ** ***n:1166***	**231 (21.8) ** ***n:1058***	**326 (27.4)** ***n:1190***	**229 (24.0)** ***n:953***	**41 (3.4)** ***n:1209***	**132 (10.4) ** ***n:1267***	**248 (20.4) ** ***n:1218***	**N/A**	**686 (54.1) ** ***n:1269***	**453 (35.7) ** ***n:1269***
*Stafilococcu* *n=10 (0.6)*	1 (16.7) *n:6*	N/A	3 **(42.9)** *n:7*	N/A	N/A	N/A	0 (0) *n:9*	N/A	N/A	0(0) *n:7*	6 **(75.0)** *n:8*	N/A	2 (**20)** *n:10*	0 (0) *n:10*
*Streptococcus* *n=15 (0.9)*	N/A	N/A	1 (6.7) *n:15*	N/A	N/A	N/A	1 (10) *n:10*	N/A	0 (0) *n:4*	N/A	N/A	N/A	0 (0) *n:15*	0 (0) *n:15*
*CNS n=28 (1.8)*	12 **(60)** *n:20*	N/A	7 **(63.6)** *n:11*	N/A	N/A	N/A	4 (16.0) *n:25*	N/A	N/A	1 (4.5) *n:22*	16 **(59.3)** *n:27*	N/A	13 **(46.4)** *n:28*	4 (14.3) *n:28*
*Serratia* *n=3 (0.2)*	3 **(100)** *n:3*	2 **(66.7)** *n:3*	N/A	1 **(100)** *n:1*	0(0) *n:2*	0 (0) *n:2*	1 **(33.3)** *n:3*	2 **(100)** *n:2*	1 **(50)** *n:2*	0(0) *n:3*	0 (0) *n:3*	N/A	2 **(66.7)** *n:3*	1 (**33.3**) *n:3*
*Providencia* *n=2 (0.1)*	2 **(100)** *n:2*	2 **(100)** *n:2*	N/A	N/A	0 (0) *n:1*	0 (0) *n:2*	1 **(50)** *n:2*	2 **(100)** *n:2*	2 **(100)** *n:2*	1 **(50)** *n:2*	1 **(50)** *n:2*	N/A	2 (**100)** *n:2*	2 (**100**) *n:2*
*Proteus* *n=26 (1.6)*	9 **(34.6)** *n:26*	3 (12.0) *n:25*	N/A	19 **(100)** *n:19*	4 (15.4) *n:26*	4 (16) *n:25*	18**(72.0)** *n:25*	3 (13.0) *n:23*	18**(72.0)** *n:25*	1 (4) *n:25*	1 (4.0) *n:25*	N/A	17 (**65.4**) *n:26*	11 (**42.3**) *n:26*
*Citrobacter* *n=8 (0.5)*	5 **(62.5)** *n:8*	3 **(42.9)** *n:7*	N/A	5 **(62.5) n***:8*	1 **(20)** *n:5*	1**(20)** *n:5*	1 (12.5) *n:8*	2 **(28.6)** *n:7*	1 (14.3) *n:7*	0 (0) *n:8*	1 (14.3) *n:7*	N/A	1 (12.5) *n:8*	1 (12.5) *n:8*
*Enterococcus* *n=43 (2.7)*	22 **(52.4)** *n:42*	N/A	N/A	N/A	N/A	N/A	N/A	N/A	4 (13.3) *n:30*	N/A	15 **(40.5)** *n:37*	3 **(100)** *n:3*	12 (**27.9**) *n:43*	2 (4.7) *n:43*
***Klebsiella*** ***n=152 (9.6)***	**134(93.1) ** ***n:144***	**58 (40) ** ***n:145***	**N/A**	**65(84.4) ** ***n:77***	**15(11.1) ** ***n:135***	**29 (23.6) ** ***n:123***	**44(30.6) ** ***n:144***	**61(58.1)** ***n:105***	**28(20.3)** ***n:138***	**20(13.2)** ***n:152***	**32 (21.3) ** ***n:150***	**N/A**	**111(73.0) ** ***n:152***	**87 (57.2) ** ***n:152***
*Pseudomonas and Acinetobacter* *n=32 (2)*	N/A	N/A	N/A	N/A	N/A	N/A	N/A	N/A	N/A	N/A	24 **(75.0)** n:30	N/A	N/A	N/A
*TOTAL*	810 (54.0) n:1499	353 (26.0) n:1357	11 (33.3) n:33	725 (45.7) n:796	179 (13.4) n:1335	265 (21.8) n:1215	396 (28.0) n:1416	299 (27.4) n:1092	95 (6.7) n:1417	155 (10.4) n:1486	344 (22.8) n:1507	3 (100) n:3	846 (54.4) n:1556	561 (36.1) n:1556

**AM**: ampicillin, **AMC**: amoxicillin-clavulanate, **P:** benzylpenicillin, **CZ**: cefazolin, **FOX**: cefoxitin, **CXA**: cefuroxime-axetil, **CFM**: cefixime, **TMP-STX**: trimethoprim sulfamethoxazole, **FF**: fosfomycin, **F**: nitrofurantoin, **GEN**: gentamicin, **CIP:** ciprofloxacin, **VA**: vankomycin, **N/A**: nonavailable. Data of *E. coli* and *Klebsiella* spp. are all in bold.

**Table 6:** Fosfomycin and nitrofurantoin resistance in MDR (+) *E. coli* strains.

*E. coli*	n (%)	Total (%)
Fosfomycin-resistant	37 (8.5)	434 (100)
Nitrofurantoin-resistant	166 (40.8)	407 (100)

MDR: multidrug resistance.

**Table 7:** Fosfomycin and nitrofurantoin resistance in lower UTI patients.

Patients with only lower UTI signs and symptoms	n (%)	Total (%)
Nitrofurantoin-resistant	46 (5.7)	805 (100)
Fosfomycin-resistant	151 (28.6)	528 (100)

UTI: urinary tract infection.

**Table 8:** Comparison of ciprofloxacin resistance in MDR (−) and MDR (+) *E. coli* and *Klebsiella* spp. strains.

*E. coli* vs. ciprofloxacin	MDR (−) n (%)	MDR (+) n (%)	p
Susceptible	743 (91.1)	227 (51.4)	**<0.001**
Resistant	33 (4.3)	215 (48.6)	
***Klebsiella*** ** spp. vs. ciprofloxacin**			
Susceptible	63 (96.9)	55 (63.2)	**<0.001**
Resistant	2 (3.1)	32 (36.8)	

MDR: multidrug resistance.

## Footnotes

1 Eurostat. Glossary_Nomenclature of territorial units for statistics (NUTS)-Statistics Explained. Website https://ec.europa.eu/eurostat/statistics-explained/index.php?title=Glossary:Nomenclature-of-territorial-units-for-statistics-(NUTS)

2 Eurostat. Statistical regions outside the EU. Website https://ec.europa.eu/eurostat/web/nuts/correspondence-tables/postcodes-and-nuts

3 Eurostat. NUTS Maps. Website http://ec.europa.eu/eurostat/web/nuts/overview
